# Comprehensive Analysis of m^6^A Regulators Characterized by the Immune Cell Infiltration in Head and Neck Squamous Cell Carcinoma to Aid Immunotherapy and Chemotherapy

**DOI:** 10.3389/fonc.2021.764798

**Published:** 2021-11-29

**Authors:** Zhiqiang Yang, Xiaoping Ming, Shuo Huang, Minlan Yang, Xuhong Zhou, Jiayu Fang

**Affiliations:** ^1^ Department of Spine Surgery and Musculoskeletal Tumor, Zhongnan Hospital of Wuhan University, Wuhan, China; ^2^ Department of Otorhinolaryngology-Head and Neck Surgery, Zhongnan Hospital of Wuhan University, Wuhan, China

**Keywords:** HNSCC, m^6^A regulator, TIME, m^6^A score, therapy

## Abstract

**Background:**

N6-Methyladenosine (m^6^A), which is a prevalent regulator of mRNA expression, has gathered increasing study interests. Though the role of m^6^A as being important in many biological processes (such as growth and proliferation of cancers) has been well documented, its potential role in tumor immune microenvironment (TIME) has rarely been analyzed.

**Methods:**

We downloaded RNA expression, single nucleotide polymorphism (SNP), and copy number variation (CNV) data from The Cancer Genome Atlas (TCGA). We then curated 21 m^6^A regulators and clustered patients into three m^6^A subtypes and m^6^A-related gene subtypes and compared them based on overall survival (OS). The combination of CIBERSORT as well as ssGSEA quantified the infiltration levels of immune cells and immune-related functions. The m^6^A scores were determined by using principal component analysis (PCA) algorithm. Furthermore, we evaluate the correlation of m^6^A regulators with immune and response to therapy.

**Results:**

Three m^6^A clusters were identified based on the TCGA-HNSCC cohort, and there were significant associations among them in overall outcomes and caner-related pathways. We found that three m^6^A clusters were consistent with three phenotypes: immune-inflamed, immune-dessert, and immune-excluded. HNSCC patients were divided into high– and low–m^6^A score groups based on the cutoff of m^6^A score. Patients with lower m^6^A score had better overall survival outcome. Further analysis indicated that patients with higher m^6^A score presented higher tumor mutation burden (TMB). In addition, patients in low–m^6^A score subgroup had high chemotherapeutics sensitivity. GEO cohort confirmed patients with low m^6^A score demonstrated significant overall survival advantages and clinical benefits. Low m^6^A score carry an increased neoantigen load, eliciting a response to immunotherapy, and its value in predicting survival outcomes of immunotherapy was also confirmed in three anti-PD-1 cohorts.

**Conclusions:**

Our study demonstrated that m^6^A regulators are closely related to TIME and the m^6^A score was an effective prognostic biomarker and predictive indicator for immunotherapy and chemotherapeutics. Comprehensive evaluation of m^6^A regulators in tumors will extend our understanding of TIME and effectively guide increasing study investigations on immunotherapy and chemotherapy strategies for HNSCC.

## Introduction

Head and neck squamous cell carcinoma (HNSCC) remains the primary cause of cancer death worldwide, with approximately 890,000 newly diagnosed cases per year (1). More than 50% of patients will present with local recurrence or node metastasis within 5 years caused by resistance to conventional treatment (2). Conventional treatments include surgery, radiotherapy, and chemotherapy based on the stage of patients, but most HNSCC exhibit weak prognosis because of the complex mechanisms whereby the RNA modifications were associated with different immune cell infiltrations.

Immunotherapy may provide significant therapeutic effects in identifying and eliminating tumor cells by activating patients’ immune defense system (3). This treatment yields new insights with unparalleled and synergistic survival benefits into multiple clinical management (4, 5). For example, inhibitors of CTLA-4 as well as anti-PD-1/L1 antibodies, which are representative immune checkpoint inhibitors, have achieved a marked clinical response in patient’s treatment (6–8). Nevertheless, a major limitation of this treatment (the imbalance of the immune system) is that a minority of patients could benefit from immunotherapy. In addition, numerous cytokines (such as IL-10 and IL-17) and immunosuppressive cells (derived from marrow) are components of the tumor immune microenvironment (TIME) promoting immune escape (9). Thus, the regulatory mechanism and the novel markers of HNSCC should be urgently investigated by comprehensively parsing the components of TIME so that the ideal HNSCC subgroups for guiding and predicting therapeutic responsiveness could be identified.

The methylation modification of the N6 adenosine (m^6^A), which is the most common type of posttranscriptional modification on RNA and mediate above 60% of RNA methylation, plays crucial roles in a series of cancer processes and progression and immunomodulatory abnormalities (10). To be specific, the aberrant methylation of m^6^A is close to cancer stem cell differentiation, cancer immune response, and microRNA (miRNA) editing; they also play an essential role in the progression of various cancers (11–13). The m^6^A methylation levels in tumors mainly depend on the expression of m^6^A regulatory proteins, which is controlled by the expression of “writers”—methyltransferases, the “erasers”—demethylases, and “readers”—binding proteins in cell (14, 15). The writers, which include methyltransferase like (METTL)14, METTL3, WT1-associated protein (WTAP), Casitas B-lineage proto-oncogene like 1 (CBLL1), KIAA1429, ZC3H13, and RNA-binding motif protein 15 (RBM15), RBM15B, promote m^6^A RNA methylation (16–18). The erasers, which include fat mass- and obesity-associated protein (FTO) and α-ketoglutarate-dependent dioxygenase alkB homolog 5 (ALKBH5), remove m^6^A methylation (19). The readers, which include YTH domain-containing 1 (YTHDC1), YTHDC2, YTH N6-methyl-adenosine RNA-binding protein 1 (YTHDF1), YTHDF2, YTHDF3, and heterogeneous nuclear ribonucleoprotein C (HNRNPC), insulin-like growth factor 2 mRNA-binding protein 2 (IGF2BP2), IGF2BP3, ELAV-like RNA-binding protein 1 (ELAVL1), heterogeneous nuclear ribonucleoprotein A2B1 (HNRNPA2B1), and LRPPRC, can bind proteins to the m^6^A methylation site (20).

Increasing evidence has demonstrated that the dysregulated expression of m^6^A regulators plays a vital regulatory role in tumor progression and patient prognosis (21, 22).

Lan et al. showed that m^6^A-modified GATA3 pre-mRNA was mediated by KIAA1429, stimulating the RNA-binding protein to undergo separation and promoting GATA3 pre-mRNA degradation (23). Among patients with hepatocellular carcinoma, overexpression of KIAA1429 was significantly associated with poor clinical prognosis. Also, shRNA silencing of KIAA1429 suppressed hepatocellular carcinoma cell proliferation and tumorigenesis both *in vitro* and *in vivo*.

In the study of Chen et al., WTAP was found to be highly expressed in osteosarcoma cancer (24), and Cox analysis showed that it was an independent prognostic factor for overall survival. Mechanistically, WTAP, as an oncogene, regulated osteosarcoma proliferation and metastasis *via* PI3K/AKT pathway *in vitro* and *in vivo*. The study of Yi et al. divided patients into two subtypes determined *via* the consensus clustering for 15 m^6^A methylation regulators, which could stratify the prognosis of patients (25). They also established the risk score based on six m^6^A regulators, which was an independent prognostic indicator of patients.

However, the role of risk score in immunotherapy and chemotherapy was not analyzed. In addition, whether m^6^A methylation regulators have the interface of copy number variations (CNVs) or the correlation of tumor mutation burden (TMB) has yet to be fully explored.

Using public databases, Li et al. showed that higher expression of METTL3 was associated with poorer survival prognosis in colorectal carcinoma (CRC) metastatic tissues (26). *In vivo*, they found that METTL3 is linked to CRC development through maintaining SOX2 expression.

Recently, most studies have revealed the correlation between immune cell infiltration and m^6^A modification, but the carcinogenic pathways of m^6^A methylation in TIME remains unclear. Han et al. reported that lysosomal protease, marked and recognized by YTHDF1, induced the degradation of tumor neoantigens (27). Compared with WT mice, they observed higher levels of CD8+ cytotoxic T cells and NK cells in tumors from YTHDF1 knockout mice, which suggest that an enhanced antitumor response occurs in the absence of YTHDF1. In melanoma cells, Chong et al. demonstrated that interferon-gamma (IFN-γ)-induced cytotoxicity could be decreased by FTO *in vitro* by suppressing the expression of cell-intrinsic genes PD-1, CXCR4, and SOX10, at least partially through YTHDF2-mediated decay process (28). Moreover, they found that knockdown of FTO enabled an antimelanoma response *via* upregulating the expression level of IFN-γ in mice. Another study demonstrated that METTL3-mediated m^6^A of CD40 and CD80 promoted DC activation and maturation, which contributed to increased antigen presentation and T-cell stimulation *in vivo* and *in vitro* (29). Also, the METTL3-mediated mRNA modification is essential in cancer progression. Consequently, these results indicated that m^6^A are vital mediators of TME, emphasizing potential promising targets in enhancing therapeutic response to clinical immunotherapy. However, almost all studies focused on one or two m^6^A regulators owing to existing technical limitations. Thus, the combined analysis of multiple m^6^A regulators in HNSCC, including the interactions between the m^6^A regulators and CNVs and TMB, will enhance our understanding of TIME (30).

In our study, we systematically assessed the relationship between m^6^A methylation and prognosis, CNVs, TMB, and TIME based on the next-generation sequencing data of HNSCC samples. Three clustering subtypes were identified *via* “ConsensusClusterPlus” method, and these three subtypes were closely linked to three phenotypes: immune-inflamed, immune-excluded, and immune-desert (7). Moreover, we constructed a scoring model, m^6^A score, to quantify HNSCC of individual cases. Also, the relationships between scoring model, ICI treatment, TIME, and cancer-related pathways were thoroughly analyzed to further explore the effect of m^6^A regulators in HNSCC. The whole study suggested that m^6^A regulators play an indispensable role in TIME and in assisting to make therapeutic strategies on HNSCC.

## Methods

### The Collection and Pretreatment of Datasets and Samples

The genomics data and clinical information of 528 HNSCC samples and 43 adjacent normal tissues were procured from the public TCGA (https://cancergenome.nih.gov/). The selection criteria were used as follows: (1) histologically confirmed HNSCC and (2) complete clinical and OS data. Lastly, 479 patients with the corresponding clinical information, including age, gender, stage, HPV subtype, and radiation therapy were collected for further analysis. The mutation data (e.g., somatic mutation and copy number variation data) was downloaded from the UCSC Xena (https://gdc.xenahubs.net/). Twenty-one m^6^A regulators were collected based on published literature. Next, the differential expression of the 21 m^6^A regulators was presented in a heatmap. Nonsynonymous mutation and synonymous mutation counts were defined as tumor mutation burden. The GSE65858 (*N* = 267) from GEO was used as the validation cohort. The detailed information of clinical data and 21 m^6^A regulators are shown in **Supplementary Tables S1**–**S3**.

### The Consensus Clustering of 21 m6A Regulators by Consensus Cluster Plus

To elucidate the biological function of the m^6^A regulators in HNSCC, ConsensusClusterPlus package based on Euclidean distance and Wards linkage was employed to classify the patients into different distinct m^6^A subtypes (31). The “PCA” package was used to investigate gene-expression arrays among distinct m^6^A subgroups.

### Gene Set Variation Analysis

We utilized the gene set variation analysis (“GSVA”) package to investigate the biological processes among different m^6^A subgroups (32). The well-defined biological pathways and functions were derived from the Hallmarker gene set “c2.cp.kegg.v7.4.symbols.gmt” and “c5.go.v7.4.symbols.gmt” (download from MSigDB database v7.4) and IMvigor210CoreBiologies package (33, 34). The “ClusterProfiler” package was used to determine the Gene Ontology (GO) annotation of m^6^A-related genes (the cutoff value were *q*-value <0.05 and *p*-value <0.05) (35).

### Immune Cell Infiltration and Immune-Related Function Estimation by ssGSEA

The relative abundance and activity levels of 23 immune cell types, obtained from published signature gene lists, were quantified using the single sample gene set enrichment analysis (ssGSEA) in R package GSVA (36, 37). In this study, the innate immune cells (including natural killer (NK) cells, CD56dim NK cells, CD56bright NK cells, dendritic cells (DCs), plasmacytoid dendritic cells (pDC), immature DCs (iDC), neutrophils, mast cells, and macrophages) and the adaptive immune cells (including B cells, T cells, CD8 T cells, T follicular helper (TFH), Th1, Th2, Th17, and Treg cells) comprised these signatures. In addition, we also used ssGSEA to explore the relationship between different m^6^A subtypes and immune-related pathways (such as cytolytic activity, T-cell co-stimulation, inflammation promoting, and parainflammation) in HNSCC expression profile of TCGA. The biosimilarity of the infiltrating immune cells and immune-related functions were estimated by the Gaussian fitting model.

### To Calculate the Immunotherapy Predictors: IPS, TIDE, and ESTIMATE

Immunophenoscore (IPS) is an effective predictor of response to immune therapy *via* characterizing the determinant factors of cancer immunogenicity and antigenomes (37). The major histocompatibility complex (MHC)-related molecules, checkpoints or immunomodulators (CP), effector cells (EC), and suppressor cells (SC) developed the IPS scoring scheme. The sum of the four classes, calculated by averaging the Z-scores, was defined as the IPS. To predict immune checkpoint blockade response (ICB), we utilized the tumor immune dysfunction and exclusion (TIDE) method to model tumor immune evasion mechanisms, including the dysfunction of T-cell dysfunction in tumors with high infiltration of cytotoxic T lymphocytes (CTLs) and the prevention of T cell in tumors with exclusion of CTLs (38). For patients with higher TIDE score, cancers more likely to occur immune escape in these patients' body, thus ICB treatment might bring these patients less and short-lasting clinical benefits. The ESTIMATE algorithm was used to evaluate the tumor cellularity and tumor purity, which were composed of the TIME, based on expression matrixes. The analysis method is integrated in the “ESTIMATE “ package (39). We extracted these gene expression data from RNASeqV2 data to predict different infiltration levels of immune cells and the proportion of stromal cells. Tumor purity is the summation of stromal score and immune score from individual cases. The tumor sample with higher immune scores and lower tumor purity indicated that it had an abundance of immune cell infiltration.

### The Identification of Significant Mutational Genes and Signatures

The mutation annotation format (maf) file was analyzed using MutSigCV algorithm to identify significant SMGs based on the significance threshold, and the maf data were processed using the “maftools” package (40). MutSigCV measures the significance of nonsilent somatic mutations in a gene based on the background mutation rates by silent mutations (41). The false discovery rates (*q*-values) were then calculated, and genes with statistical significance (*q*-values ≤0.1) were set as SMGs (**Supplementary Table S4**). We then utilized the waterfall plot to visualize the mutation information of these significant SMGs in the TCGA cohort. Furthermore, we applied Fisher’s test to detect mutually exclusive or co-occurring ratio of m^6^A regulators. Mutational signatures were determined using the genomic data by adopting ExtractSignatures function that applies the Bayesian nonnegative matrix factorization-based framework (42). The optimal number of mutational signatures for the TCGA cohort could be detected by the SignatureEnrichment function and then it automatically assigned a given signature to each sample.

### DEGs Associated With the m6A Phenotypes

Patients were grouped into the three m^6^A clusters based on consensus clustering algorithm to identify differentially expressed genes (DEGs) associated with the m^6^A modification. The “limma” package was implemented to determine DEGs between three m^6^A clusters (43). The significance filtering cutoff of DEGs were set as the significance-adjusted *p*-value <0.001.

### The Construction of the m6A Gene Signature

The overlapped DEGs identified from DEGs were used to perform the univariate Cox regression. The consensus clustering algorithm was utilized to define the number of gene clusters. The prognosis-related genes were extracted for further analysis. We then curated the final genes determined to conduct principal component analysis (PCA), and principal component 1 and 2 were extracted to construct the m^6^A score (44, 45). This method has an advantage of mainly focusing on positively correlated (or negatively correlated) genes. We then define the m^6^A score of each patient by adopting a similar formula based on the previous studies:


$${\text{m}}^{6}\text{A score}=\sum \left(\text{PC}1_{\text{i}}\right)+\sum \left(\text{PC}2_{\text{i}}\right)$$

To determine the TMB of each patient, we also counted the nonsynonymous and synonymous mutation counts in the TCGA cohort (46). The association with TMB and m^6^A score was evaluated by Spearman’s method based on survival curve.

### The Correlation Between m6A Score and Biological Pathways

Mariathasan et al. constructed a panel of signatures that stored genes associated with various biological pathways, including (1) immune-checkpoint; (2) CD8 T-effector signature; (3) epithelial-mesenchymal transition (EMT), including EMT1, EMT2, and EMT3; (4) pan-fibroblast TGFb response signature (Pan-F-TBRS); (5) Fanconi anemia pathway; (6) homologous recombination; (7) base excision repair; (8) WNT target; (9) DNA damage repair; (10) mismatch repair; (11) nucleotide excision repair; (12) DNA replication; (13) antigen processing; (14) cell cycle regulation; (15) FGFR3-related genes; and (16) cell cycle (34, 47). We performed the Spearman’s method to explore the correlation between m^6^A score and these biological pathways.

### The Genomic and Clinical Information of Immune-Checkpoint Cohorts

We systematically performed a search for the ICB cohorts in the public databases, which could be available for detailed genomic and clinical information. Three independent anti-PD-L1 cohorts, IMvigor210 cohort (patients with metastatic urothelial cancer treated with atezolizumab) (34), Riaz et al. cohort (patients with metastatic melanoma treated with nivolumab) (48), and GSE78220 cohort (patients with metastatic melanoma treated with pembrolizumab) (49), were finally downloaded to analyze the predictive value of the m^6^A score for immunotherapy. The raw gene expression data of all cohorts were normalized.

### To Evaluate the Sensitivity of Chemotherapeutic Drugs

We used the largest public pharmacogenomics database, Genomics of Drug Sensitivity in Cancer (GDSC), to predict the sensitivity of different drugs between high– and low–m^6^A score subgroups (50). The prediction process used was the “pRRophetic” package where the half-maximal inhibitory concentration (IC_50_) was estimated by ridge regression model based on gene expression profiles (51).

### Statistical Analyses

The statistical analyses were generated by using R version 4.1.0. To compare more than two groups, statistical significance was estimated by the Kruskal-Wallis test. Student’s *t*-test was used to compare the difference between two subgroups (52). Kaplan-Meier analysis generated the differences between m^6^A subgroups and prognosis *via* the “survminer” package. To determine the optimal cutoff values of each cohort, we used the “surv-cutpoint” function from the “survival” package. We adopted Cox regression to calculate the hazard ratios (HR) of m^6^A regulators and m^6^A-related genes. The multivariate Cox regression was used to evaluate the independent prognostic factors. The “forestplot” package was employed to show the results of Cox regression analysis for m^6^A score in the GEO cohort and TCGA cohort. We assessed the specificity and sensitivity of m^6^A score through drawing receiver operating characteristic (ROC) curve by using “pROC” and “‘timeROC” package. Also, the Spearman’s method was used to compute the correlation coefficient. All comparisons were presented by the *p*-values (two-tailed), whereby <0.05 indicated statistical significance.

## Results

### The Genetic Landscape of 21 m6A Regulators in HNSCC

**Figure 1:**
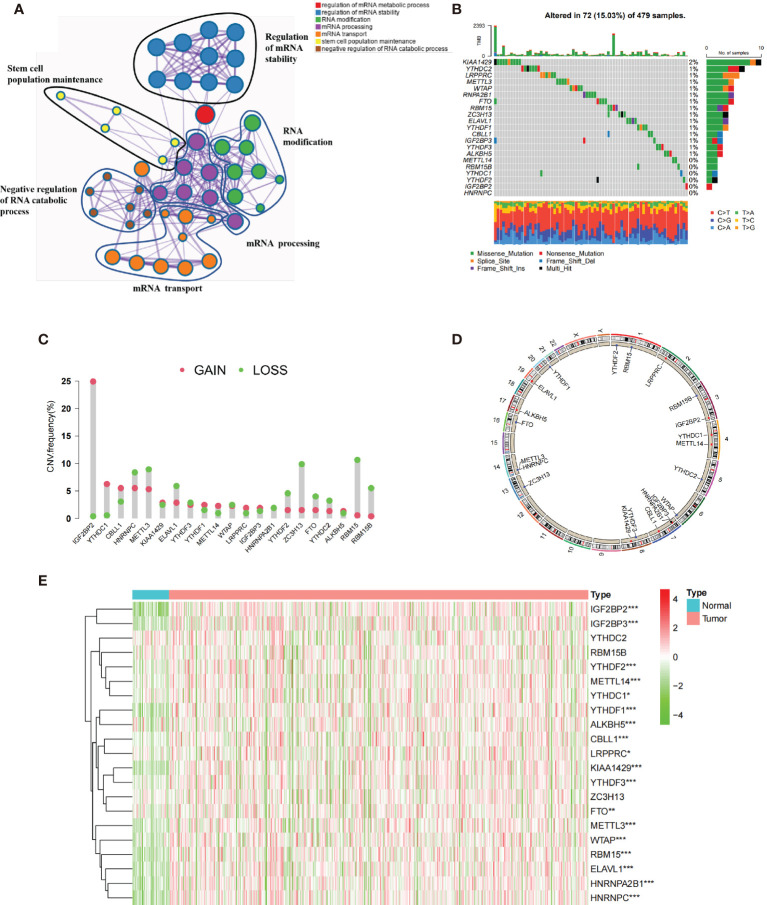
The genetic landscape of 21 m^6^A regulators in HNSCC. **(A)** The functional enrichment network of 21 m^6^A regulators visualized by Metascape. Different circles represented different annotations. **(B)** Seventy-two of the 479 patients showed different genetic alterations, including missense mutation, splice site, and nonsense mutation. **(C)** The CNV of 21 m^6^A regulators. The column represented the alteration frequency. The green dots represented deletion of CNV. The pink dots represented amplification of CNV. **(D)** The location of CNV alteration of m^6^A regulators in cell. **(E)** The different expression level of 21 m^6^A regulators between normal and HNSCC (^*^ *p* < 0.05; ^**^ *p* < 0.01; ^***^ *p* < 0.001).

We firstly identified 21 m^6^A regulators (including eight “writers,” 11 “readers,” and two “erasers”) in the TCGA cohort. **Figure 1A** and **Supplementary Figure S1A** summarize the significant biological processes and functions of 21 m^6^A regulators conducted by Metascape database. Then, the waterfall plot presented the incidence of copy number variations and the ratio of somatic mutations of 21 m^6^A regulators. A total of 72 of the 479 (15.03%) patients experienced mutations, mainly including missense mutation, splice site, and nonsense mutation. In **Figure 1B**, we found that KIAA1429 exhibited the highest mutation frequency, followed by LRPPRC and YTHDC2, while YTHDC1, YTHDF2, IGF2BP2, HNRNPC, METTL14, and RBM15B did not show any mutations. The results of mutation co-occurrence examined the significant relationship between IGF2BP3 and FTO, RBM15 and YTHDF1, LRPPRC and YTHDF2 (**Supplementary Figure S1B**). Further investigation revealed the CNV frequency of 21 m^6^A regulators. Most m^6^A regulators showed the prevalent deletions in copy number, while IGF2BP2, YTHDC1, and CBLL1 had a widespread frequency of CNV amplification (**Figure 1C**). **Figure 1D** shows the location of CNV of all m^6^A regulators on chromosomes. We further demonstrated that the expressions of ALKBH5, METTL3, YTHDF2, and YTHDC2 were significantly downregulated in tumor samples, and in contrast the expression of CBLL1, METTL14, IGF2BP2, IGF2BP3, KIAA1429, YTHDF1, and YTHDC1 were significantly upregulated in tumor samples (**Figure 1E**). Compared with normal tissues, m^6^A regulators (such as CBLL1 and YTHDF1) with amplificated CNV demonstrated markedly higher expression, and YTHDF2 and YTHDC2 with prevalent CNV deletions were markedly decreased in the tumor (**Figures 1C, E**). Spearman’s method presented the correlation among these m^6^A regulators (**Supplementary Figure S1C**). We found that IGF2BP2 showed no significant correlation with some m^6^A regulators (RBM15B, YTHDC2, RBM15, YTHDF2, and METTL14). We then ascertain the prognostic value of 21 m^6^A regulators using the Cox regression. The Cox regression revealed that YTHDC2 was a protective factor, significantly associated with prolonged overall survival rate, while HNRNPA2B1 was a risk factor (**Supplementary Figures S1D, E**). Based on these results, we demonstrated that m^6^A regulators had significant heterogeneity of genomic and transcriptomic alteration landscape between normal and HNSCC samples.

### The Identification of m6A Subgroups Mediated by 21 m6A Regulators

**Figure 2:**
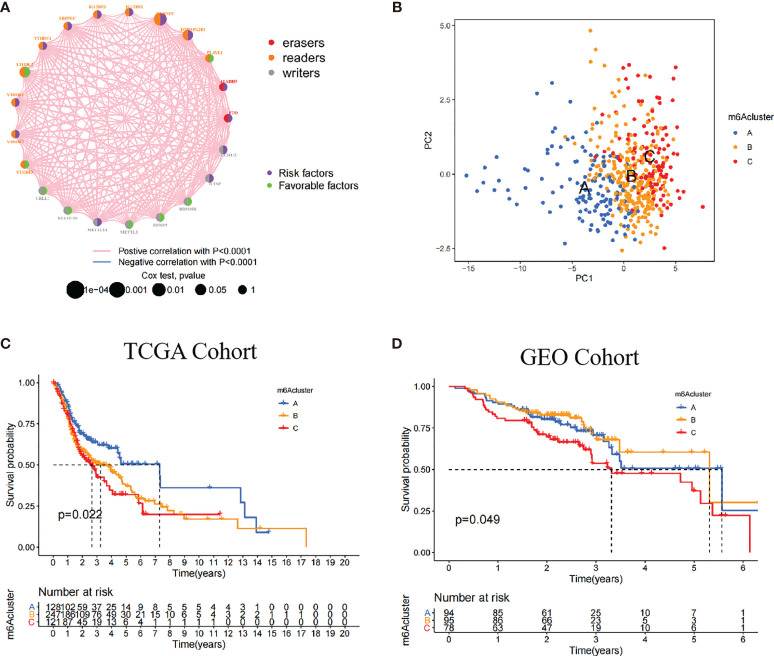
Patterns of m^6^A methylation modification. **(A)** The interaction of 21 m^6^A regulators in HNSCC. The different RNA modifications were depicted by different colored circles. Readers, orange; writers, gray; erasers, red. Favorable factors were indicated by the green circle, and risk factors were indicated by the purple circle. **(B)** The remarkable difference between different three m^6^A clusters was plotted *via* principal component analysis. **(C)** Kaplan-Meier curves of overall survival (OS) in TCGA cohort with three m^6^A clusters (*p* = 0.022). **(D)** Kaplan-Meier curves of overall survival (OS) in GEO cohort with three m^6^A clusters (*p* = 0.049). The patients in m^6^A cluster C showed worse OS than in other clusters.

The TCGA dataset with available survival and clinical information were enrolled into the training cohort. The regulator network comprehensively depicted the whole interactions of 21 m^6^A regulators and their prognostic significance (**Figure 2A**). We found that not only eraser genes were all risk factors, while some of the writer and reader genes were favorable factors. Moreover, we demonstrated that the connection among 21 m^6^A regulators were positively correlated. These results indicated that cross-talk among the 21 regulators probably play critical roles in the formation of different m^6^A modifications and pathogenesis and progression in individual tumors. Based on the hypotheses, we utilized unsupervised clustering to classify samples into different m^6^A clusters. Moreover, we could completely distinguish one m^6^A cluster from other clusters based on PCA (**Figure 2B**). Accordingly, three distinct m^6^A clusters were eventually identified, including 128 cases in m^6^A cluster A, 247 cases in m^6^A cluster B, and 121 cases in m^6^A cluster C (**Figure 2C**; **Supplementary Figures S2A, B**).

Among these clusters, m^6^A cluster A, m^6^A cluster B, and m^6^A cluster C, patients in m^6^A cluster A had an advantage in overall survival rate, whereas m^6^A cluster C revealed the poorer prognosis in the TCGA cohort (*p* = 0.022). In the validation cohort (GEO cohort), the identical analyses obtained similar results (*p* = 0.049, **Figure 2D**; **Supplementary Figure S2C**).

In the TCGA cohort, multivariate Cox regression further demonstrated that patients in m^6^A cluster C had worst overall survival rate after adjusting clinical parameters [m^6^A cluster C *vs*. m^6^A cluster A, HR, 1.68 (95% CI, 1 to 2.8), *p* = 0.049, **Supplementary Figure S4A**]. However, there was no statistical significance between m^6^A cluster C and prognostic outcome in the GEO cohort [m^6^A cluster C *vs*. m^6^A cluster A, HR, 1.47 (95% CI, 0.88 to 2.47), *p* = 0.143, **Supplementary Figure S4B**]. We also noticed that the 21 m^6^A regulators showed different significances between the three m^6^A clusters. In detail, KIAA1429 and FTO were significantly elevated in m^6^A cluster A; CBLL1, IGF2BP2, and IGF2BP3 were significantly elevated in m^6^A cluster B; and WTAP, ALKBH5, and RBM15 were significantly elevated in m^6^A cluster C (**Supplementary Figures S2B, C**).

### The Distinct Immune Landscapes of TIME in m6A Clusters

To explore the biological functions and pathways underlying these m^6^A clusters, we performed GSVA enrichment analysis against the GO and KEGG gene sets (**Supplementary Figures S3A, B**). As shown in the GSVA analysis, m^6^A cluster A was markedly enriched in immune activation-related pathways. Intriguingly, m^6^A cluster C was markedly associated with carcinogenic pathways, such as DNA replication, nucleotide excision repair, and mismatch repair pathways. Whereas, m^6^A cluster B was highly enriched in both carcinogenic and stromal-related signaling pathways.

**Figure 3:**
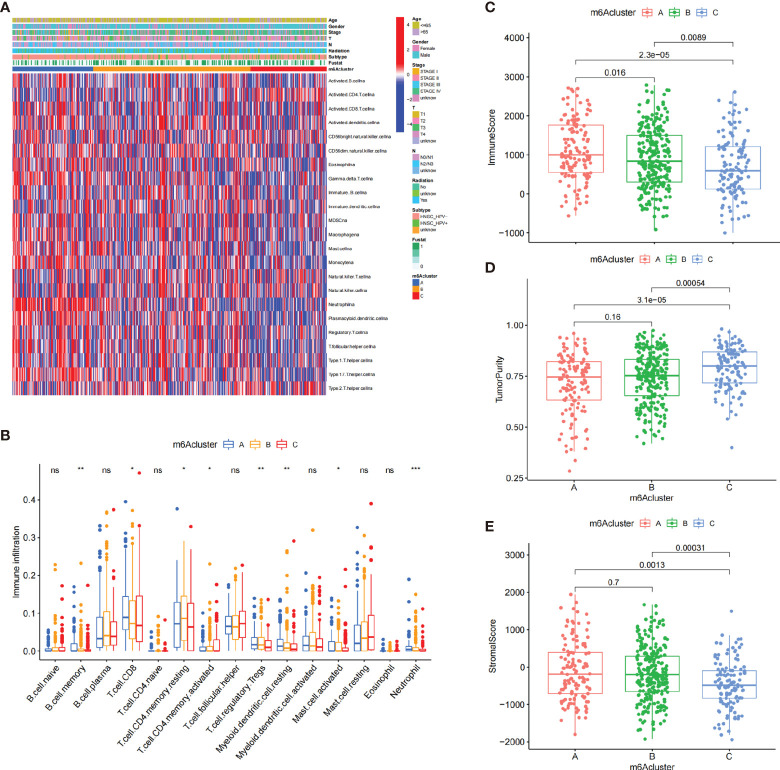
The characteristics of TIME in three m^6^A clusters. **(A)** The heatmap showed the result of the consensus clustering in the TCGA cohort. Clinical information included age, gender, survival status, HPV subtypes, radiation, and stage. **(B)** The infiltration of immune cells in the three m^6^A clusters using the CIBERSORT. ^*^ *p* < 0.05; ^**^ *p* < 0.01; ^***^ *p* < 0.001. **(C–E)** The analysis of the immune score **(C)**, tumor purity **(D)**, and stromal score **(E)** among three m^6^A clusters. ns, no significance.

The heatmap visualized the infiltration levels of 23 immune cells among three m^6^A clusters (**Figure 3A**). Antitumor lymphocyte cells, such as activated CD8+ T cells, and NK cells, were mainly enriched in the m^6^A cluster A. However, regulatory T cells and type 1/2/17 T helper cells were mainly enriched in the m^6^A cluster B. To our surprise, innate immune cells including natural killer cell, macrophage, eosinophil, mast cell, and MDSC were increased in the m^6^A cluster C. To explore the subsets of immune cell in TIME, CIBERSORT package was further used to characterize the immune cell infiltration based on the expression file. We observed the consistent result in the **Figure 3B**. Previous studies revealed a novel immune phenotype, immune-excluded phenotype, with an abundance of immune cells, retained in the tumor stroma rather than in the parenchyma. Therefore, we speculated that the m^6^A cluster B with higher stromal score exhibited an ineffective antitumor immune response (**Figure 3E**). Cancer-related pathway analyses demonstrated that the m^6^A cluster B was related to TGF-β and WNT-target pathways, which further corroborated with our hypothesis (**Supplementary Figures S4C, D**). In **Figure 3C**, we found that m^6^A cluster A exhibited the highest immune scores, followed by m^6^A cluster B and m^6^A cluster C. Conversely, m^6^A cluster C had a higher tumor purity than m^6^A cluster B and m^6^A cluster A, suggesting that tumors in m^6^A cluster B and m^6^A cluster A are surrounded by more immune cells and stromal cells (**Figure 3D**).

Then, we examined the association between 21 m^6^A regulators and immune cells *via* Spearman’s method. We focused on the regulator HNRNPA2B1, an independent prognostic risk factor based on the above results (**Supplementary Figures S1D, E**), which was negatively correlated with numerous immune cells (**Supplementary Figure S5A**). The ESTIMATE showed that low-expression subgroup of HNRNPA2B1 exhibited higher immune score, which confirmed the above findings (**Supplementary Figure S5B**).

We also found that low-expression subgroup of HNRNPA2B1 exhibited a significant increased among 23 immune cells (**Supplementary Figure S5C**). The low-expression subgroup of HNRNPA2B1 also exhibited elevated expression of HLA molecules (**Supplementary Figure S5D**). Subsequent function enrichment analyses found that low-expression subgroup of HNRNPA2B1 exhibited an obvious enhancement in immune activation including T-cell costimulation and type I/II IFN responses, which hinted that the expression of HNRNPA2B1 might affect the efficacy of immunotherapy (**Supplementary Figure S5D**). Thus, we investigated two anti-PD-L1 immunotherapy cohorts (IMvigor210 cohort and GSE78220 cohort). In the IMvigor210 cohort, patients with low expression of HNRNPA2B1 had prolonged overall survival rate (**Supplementary Figure S5E**). In the GSE78220 cohort, there was no significant survival trend (**Supplementary Figure S5F**). Therefore, we speculated that HNRNPA2B1-mediated m^6^A methylation modification may enhance the antitumor response *via* promoting the activation of immune cells.

### The m6A-Related DEGs in HNCSS

**Figure 4:**
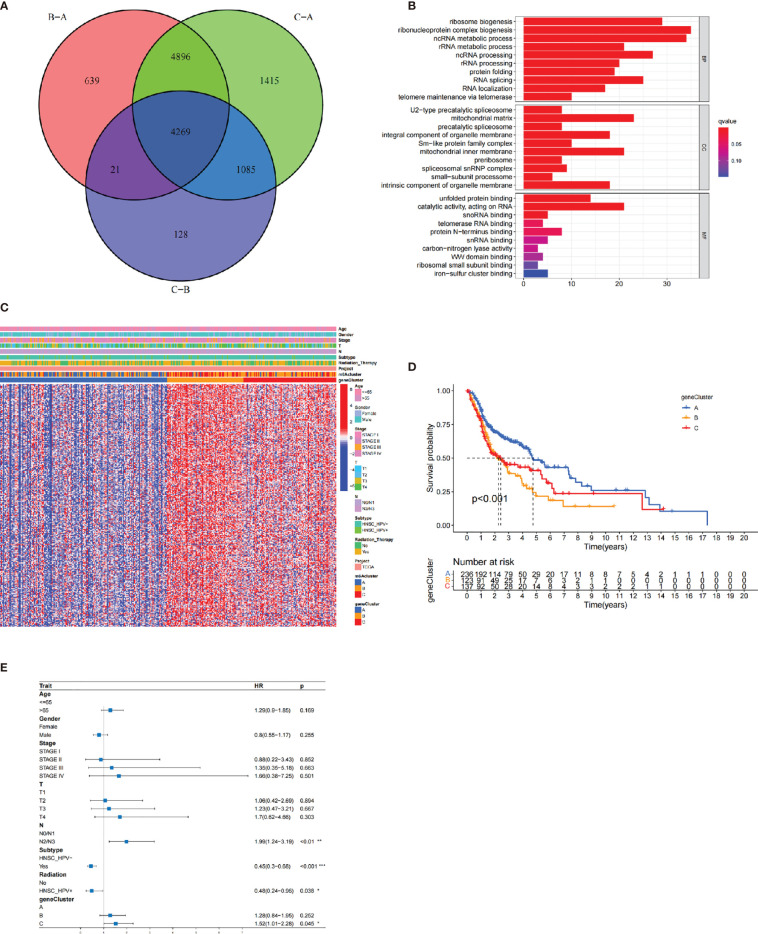
The construction of m^6^A gene clusters and functional annotation. The 4,269 differentially expressed genes (DEGs) among three m^6^A clusters were shown in the Venn plot. **(B)** GO enrichment analysis of 311 prognosis-related DEGs. **(C)** The consensus clustering based on prognosis-related DEGs classified patients into three gene clusters, respectively. **(D)** The Kaplan-Meier curves of the three m^6^A gene clusters (*p* < 0.001). **(E)** The multivariate Cox regression-estimated prognostic value of m^6^A gene clusters in TCGA cohort.

To identify the biological behaviors (e.g., genetic alterations and expression perturbations) of these m^6^A clusters, we fixed attention on the m^6^A-related transcriptional expression alterations across three m^6^A clusters in HNSCC. The Venn diagram determined 4,269 overlapping differentially expressed genes (DEGs) (**Figure 4A**). A total of 311 DEGs related to prognosis were considered the representative m^6^A-related genes (**Supplementary Table S5**). GO enrichment analysis revealed that the biological processes related to RNA transcription and modification were significant functions (**Figure 4B**). Similar to the above analysis, unsupervised clustering method based on the expression of these 311 DEGs separated patients into three stable gene clusters (gene clusters A–C) in the TCGA cohort (**Supplementary Figure S6A**). **Figure 4C** demonstrates that three m^6^A gene cluster had different clinicopathological features. We found that patients in m^6^A gene cluster C exhibited advanced clinical stage. In addition, patients receiving radiotherapy were mainly concentrated in the m^6^A gene cluster A, while patients with negative HPV subtype were represented by the m^6^A gene clusters B and C.

The survival analysis further indicated that the three m^6^A gene clusters had significant prognostic differences in HNSCC samples. m^6^A gene cluster A was proven to be related to better prognostic outcome, while patients in m^6^A gene cluster C was associated with poorer outcome (**Figure 4D**). The Cox regression determined m^6^A gene cluster C (*vs*. m^6^A gene cluster A) as an independent risk factor after considering age, gender, stage, HPV subtype, and radiotherapy [HR, 1.52 (95% CI, 1.01 to 2.28), *p* = 0.045; **Figure 4E**]. **Supplementary Figure S6B** observes the different expression levels of the 21 m^6^A regulators, which were consistent with our expected results.

### The Construction of Prognostic Signatures and Exploration of Its Characteristics of Clinical Traits

Accordingly, the above results showed that the m^6^A regulators played a nonnegligible role in regulating prognosis and TIME. However, these analyses were only based on the overall population and could not interpret the heterogeneity and complexity of m^6^A regulators individually. Based on these identified m^6^A-related genes, we developed a scoring scheme, considered m^6^Ascore, to quantify individual patients.

**Figure 5:**
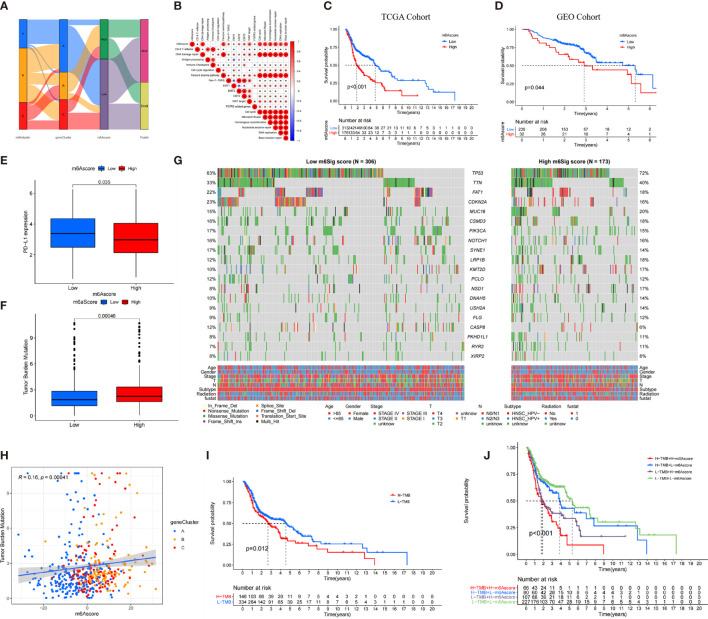
The construction of m6A score and explore its relevant genetic features. **(A)** Alluvial diagram of m6A clusters in groups with m6A geneCluster, m6A score, and survival status. **(B)** Correlations between m6A score and the known biological gene signatures using Spearman analysis. **(C, D)** The survival analysis of patients in high and low m6A score subgroups in the TCGA cohort **(C)** and GEO cohort **(D)**. **(E)** Comparison of PD-L1 expression level in high versus low m6A score subgroups. **(F)** The distribution of tumor mutation load (TMB) in high versus low m6A score subgroups. **(G)** The waterfall of mutational landscape in TCGA stratified by low (left panel) and high m6Sig score (right panel) subgroups. Each column represented one patient. Age, Gender, Survival status, HPV subtypes, Radiation, Stage were shown as patient annotations. **(H)** There was a significant positive correlation between the m6A score and TMB (R = 0.16, p < 0.001). **(I)** Kaplan-Meier curves for patients in high and low TMB subgroups. H, high; L, Low (P=0.012). **(J)** Kaplan-Meier curves for patient in the TCGA cohort stratified by both m6A score and TMB. H, high; L, Low; TMB, tumor mutation load (P < 0.001).

The alluvial diagram visualized the quantification changes of patients (**Figure 5A**). These results illustrated that m^6^A gene clusters B and C were linked to higher m^6^A score, whereas m^6^A gene cluster A exhibited lower m^6^A score. Notably, m^6^A cluster C showed the highest m^6^A score, followed by m^6^A cluster B, while m^6^A cluster A revealed the lowest m^6^A score (**Supplementary Figure S7A**). Furthermore, we conducted the analysis of Spearman’s correlation to illustrate the patterns of m^6^A regulators. The heatmap indicated that m^6^A score was positively correlated with WNT target signatures, cell cycle signatures, and EMT pathways (**Figure 5B**).

There was an inverse trend between the m^6^A score and the immune score (*R* = −0.35, *p* = 2.3e−16) and stromal score (*R* = −0.08, *p* = 0.076), which demonstrated the crosstalk between m^6^A score and TIME (**Supplementary Figure S7B, C**). Compared with the high m^6^A score, patients in low–m^6^A score subgroup had higher relative level of immune checkpoint molecules and CD8 effector cells. However, high m^6^A scores were significantly associated with stromal pathways (**Supplementary Figure S7D**).

Furthermore, we determined the prognostic value of m^6^A score in predicting patients’ survival outcome. Based on the cutoff value of 3.3615, we divided patients into low– or high–m^6^A score subgroups. As expected, patients with low–m^6^A score were associated with a prominent prognosis (*p* < 0.001, **Figure 5C**), and the ROC validated the predictive accuracy of the m^6^A score (AUC = 0.634, **Supplementary Figure S7E**). Integrating the clinical information (e.g., age, gender, stage, HPV subtype, and radiotherapy), multivariate Cox regression confirmed that the high m^6^A score was an independent prognostic factor for evaluating survival outcome (high m^6^A score *vs*. low m^6^A score; HR, 0.61 [95% CI, 0.44 to 0.86], *p* < 0.01, **Supplementary Figure S7G**).

We also investigated the relationship between the m^6^A score and level and found that the expression level of PD-L1 was elevated in the low–m^6^A score subgroup than in the high m^6^A score subgroup (**Figure 5E**). The constructed m^6^A score was validated in the GEO cohort by integrating clinical genomic information. The m^6^A score displayed the potential predictive value in GEO cohort (AUC = 0.672, **Supplementary Figure S7F**), and patients in low–m^6^A score subgroup had a better survival outcome (*p* = 0.044; **Figure 5D**). Multivariate Cox regression also confirmed that the m^6^A score was an independent prognostic biomarker in GEO cohort [HR, 0.52 (95% CI, 0.3 to 0.92), *p* = 0.024; **Supplementary Figure S7H**]. We then further analyzed the distribution of somatic mutated gene between low– and high–m^6^A score subgroups. As shown in **Figure 5G**, high m^6^A score subgroup presented more tumor somatic mutations than the low–m^6^A score group. Increasing studies have demonstrated and there was a link between the TMB and immunotherapy responses. Consequently, we further explored the distribution of TMB in low– and high–m^6^A score subgroups. We confirmed that the low–m^6^A score group had lower TMB frequencies (**Figure 5F**). The m^6^A score was markedly positively correlated with TMB (*R* = 0.16, *p* = 0.00041; **Figure 5H**). In addition, we found that patients with low TMB frequencies demonstrated a survival benefit (*p* = 0.012; **Figure 5I**), while patients with low m^6^A score showed significant survival advantages among patients with low TMB frequencies (**Figure 5J**).

The prognostic value of m^6^A score subjected to various clinical parameters was also estimated. We found that patients in low m^6^A score had a better survival outcome than those in m^6^A score among different subgroups (**Supplementary Figure S8**). Furthermore, the OS of patients with radiotherapy in the high– and low–m^6^A score groups was superior, but patients with low m^6^A score benefited significantly more than those with high m^6^A score from radiotherapy. Accordingly, patients with low m^6^A score were more likely to benefit for survival from radiotherapy than those with high m^6^A score.

### The Role of m^6^A Score in Predicting Immunotherapy Benefits

TIDE and IPS served as novel imunotherapeutic predictors and are strongly suggested to evaluate the response of immunotherapy to patients. We revealed that TIDE was significantly decreased in the low–m^6^A score subgroup, and IPS was significantly elevated in the low–m^6^A score subgroup (IPS: *p* = 0.0014, **Supplementary Figure S9A**; TIDE: *p* = 0.0035, **Supplementary Figure S9B**). In detail, the levels of the four groups were significantly increased in the low m^6^A score group (**Supplementary Figures S9C–F**).

**Figure 6:**
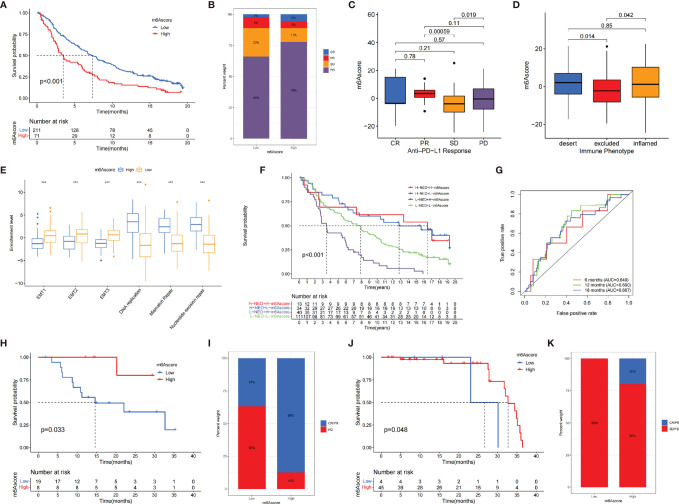
The role of m^6^A score in anti-PD-1/L1 cohorts. **(A)** The survival analysis of patients in low and high m^6^A score subgroups in the IMvigor210 cohort (*p* < 0.001). **(B)** The proportion of patients with different response to immunotherapy in IMvigor210 cohort. SD, stable disease; PD, progressive disease; CR, complete response; PR, partial response. **(C)** Distribution of m^6^A score in different response groups. **(D)** The tumor immune phenotypes were validated in IMvigor210 cohort. **(E)** Differences in EMT pathways and DNA repair-related pathways between low and high m^6^A score groups (^*^ *p* < 0.05; ^**^ *p* < 0.01; ^***^ *p* < 0.001). **(F)** The survival analysis of patients who received immunotherapy stratified by both m^6^A score and neoantigen burden. H, high; L, low; NEO, neoantigen burden (*p* < 0.001). **(G)** The AUC of the quantification of m^6^A score in patients treated with immunotherapy (6 months, AUC = 0.649; 12 months, AUC = 0.690; 18 months, AUC = 0.667). **(H)** The survival analysis of patients in low– and high–m^6^A score subgroups in the GSE78220 cohort (*p* = 0.033). **(I)** The proportion of patients with different responses to immunotherapy in GSE78220 cohort. **(J)** The survival analysis of patients in low– and high–m^6^A score subgroups in the Riaz et al. cohort (*p* = 0.048). **(K)** The proportion of patients with different immunotherapy response in Riaz et al. cohort.

We investigated whether the m^6^A score could predict immunotherapy response to ICB treatment based on three cohorts. Among IMvigor210 cohort and Riaz et al. cohort, patients with low m^6^A score exhibited clinical benefits markedly (IMvigor210 cohort, *p* < 0.001, **Figure 6A**; Riaz et al. cohort, *p* = 0.048, **Figure 6J**). In GSE78220 cohort, the survival curve presented an opposite result due to the small number of samples (**Figure 6H**). The immunotherapeutic advantages and anti-PD-1/L1 response to patients were confirmed in the low– and high–m^6^A score subgroups (**Figures 6B, C, I, K**).

We investigated the difference between m^6^A score and three immune phenotypes in the IMvigor210 cohort and found that lower m^6^A score was remarkably associated with excluded immune phenotype, indicating that immune checkpoint inhibitors are less effective for these patients (**Figure 6D**). Furthermore, we revealed that EMT were significantly activated in tumors with low m^6^A score (**Figure 6E**). **Figure 6F** indicates that individuals with a combination of high m^6^A score and low neoantigen burden showed a poorer prognosis. The ROC curves implied that m^6^A score was a robust biomarker to assess clinical prognosis of patients under immunotherapy (**Figure 6G**). In summary, our work strongly demonstrated that m^6^A regulators was significantly correlated with TIME and mediated prognostic response to immunotherapy.

### The Low–m^6^A Score Group Showed More Sensitivity to Chemotherapies

Considering the frequent use of chemotherapy in the treatment of HNSCC, we further explored the response of patients with 138 different types of drugs. In detail, the GDSC dataset was used to predict the IC_50_ of the selected drugs based on the “pRRophetic” package. A total of 54 drugs demonstrated obviously lower IC_50_ in the low–m^6^A score group (**Supplementary Figure S10**). Based on the guidelines of the National Comprehensive Cancer Network (53) and Chinese Society of Clinical Oncology (54), we summarized all the drugs used for the treatment of head and neck tumor (including cisplatin, methotrexate, cetuximab, afatinib, capecitabine, oxaliplatin, carboplatin, docetaxel, nivolumab, camrelizumab, gemcitabine, nimotuzumab, 5-FU, paclitaxel, pembrolizumab, toripalimab, and nedaplatin). However, only paclitaxel presented obvious lower IC_50_ in the low–m^6^A score group. The finding suggested that patients with low m^6^A score were more sensitive to the treatment of paclitaxel than those with high m^6^A score in HNSCC.

## Discussion

Increasing evidence shows that m^6^A methylation, the most common posttranscriptional modification, exerts a crucial regulation on immunity, inflammation, as well as antitumor effects involving in interaction with various m^6^A regulators. Furthermore, since most studies just revealed the modulation of one or two regulators in the contexture of TIME, the comprehensive characteristics of immune cell mediated by integrated various m^6^A regulators is essential to elucidate the potential mechanism of m^6^A methylation in TIME. So far, the effects of m^6^A regulators on the TIME of HNSCC have not been explained comprehensively. Identifying the contribution of m^6^A regulators in TIME will enhance our understanding of antitumor response mediated by m^6^A methylation and facilitating more effective strategies on immunotherapy and chemotherapy.

In our study, we established three immune phenotypes based on 21 m^6^A regulators, which were correlated with survival outcomes and diverse TIME characterization in HNSCC. The m^6^A cluster A had high infiltration level of adaptive immune cells, corresponding to the immune-inflamed phenotype. The m6A cluster B had high infiltration level of innate immune cells and stroma cells, corresponding to the immune-excluded phenotype. The m^6^A cluster C was characterized by the inhibition of TIME, corresponding to the immune-desert phenotype. The immune-inflamed phenotype showed a large infiltration proportion of immune cell TIME (7). The immune excluded, known as nonhot tumors, means that immune cells were penetrated in the stroma rather than parenchyma. In our study, we found that the immune-desert phenotypes lacked activated and priming T cell, which were correlated with the immune escape demonstrated by previous studies (34, 55, 56). We also revealed that the m^6^A cluster A was significantly associated with elevated infiltration of lymphocyte, supporting its predictive value on immunotherapy. Based on the above results, we found m6A cluster B exhibited a significantly high level of stroma activation, including Wnt signaling pathway and TGF-β pathway, which impeded the activation of T-lymphocyte cells (57). Therefore, we presumed that patients in m^6^A cluster B may benefit from ICB treatment as well as TGF-β blockade treatment.

The overlapped DEGs identified from three m^6^A phenotype were significantly associated with RNA modification and immune-related pathways, suggesting that these DEGs were “true” m^6^A-related genes. We then further identified three transcriptomic subtypes based on m^6^A-related genes. This result demonstrated that all m^6^A regulators played a key role in shaping TIME. After, we established a scoring system, named m^6^A score, to distinguish heterogeneity of each patient derived from m^6^A modification, thus precisely guiding therapeutic strategies for HNSCC. As observed, the m^6^A modification pattern characterized by the immune-desert phenotype exhibited a higher m^6^A score, while the pattern characterized by the immune-inflamed phenotype showed a lower m^6^A score.

Further analyses showed that the m^6^A score could serve as a prognostic biomarker, which was also associated with mutation-related signatures and TMB. These results suggested that the m^6^A score could be a preferable marker in predicting genomic aberrations.

We verified that the m^6^A score was strongly associated with the predictors of ICB treatment, implying that the m^6^A methylation could affect the response of immunotherapy to patients. In the IMvigor210 cohort, we validated the accuracy of the determined immune phenotype (34) and found that the m^6^A score integrated with various biomarkers (e.g., neoantigen load, TMB, the components of TIME) could more effectively predict prognosis of patients receiving immunotherapy. Actually, we also confirmed the prediction ability of the m^6^A score in the anti-PD-1/L1 immune response *via* two independent immunotherapy cohorts, which showed significant difference between nonresponders partial responders, and completed responders. We further found that patients with low m^6^A score might be more sensitive to anticancer drugs than high m^6^A score based on the GDSC. These above findings suggested that m^6^A score was a reliable tool, which could be used to comprehensively determine the immune-related phenotypes and guide clinical treatment decision to immunotherapy and anticancer drugs.

Furthermore, we also elucidated the specific m^6^A regulators in the regulation of TIME. Recent studies have confirmed that m^6^A could enhance the stability of mRNA and transport the specific mRNAs to the cytoplasm mainly through the binding proteins of HNRNPA2B1 in cell (58). Also, HNRNPA2B1 was recognized as an oncogene as it promotes tumor growth and migration in various cancers (59–61). Our analyses revealed that the expression of HNRNPA2B1 was upregulated in tumor and associated with decreased survival rate. Furthermore, higher expression of HNRNPA2B1 exhibited a lower infiltration trend of various types of DC, indicating that HNRNPA2B1 may be involved in the activation of DC. We also evaluated the mutated driver genes, the critical foundation of tumor diagnosis, therapeutic selections, *via* analyzing the TCGA cohort.

Although 21 m^6^A regulators are added into the mode, novel-identified regulators need to be curated to optimize the accuracy of the m^6^A score. Since there is a lack of appropriate immunotherapy cohorts based on HNSCC, we hope that different regimens (e.g., anti-PD-1/L1 or anti-CTLA-4) across HNSCC cohorts will verify our conclusion. Furthermore, only retrospective datasets were used to identify the m^6^A regulators and m^6^A score; thus, a series of prospective cohorts receiving immunotherapy were needed. Moreover, as not all cohorts exhibited that patients in low–m^6^A score subgroup benefits from ICB treatment, we needed a large and multicenter clinical population sample combined with more clinical features to confirm and improve the accuracy of the model.

In conclusion, our work comprehensively evaluated the TIME characteristics of m^6^A regulators based on different cohorts. This integrated analysis indicated m^6^A modification could not be ignored as its vital role in regulating tumor immunity. Comprehensive evaluation of m^6^A modification in TIME will guide more effective and important immunotherapeutic strategies.

## Data Availability Statement

Publicly available datasets were analyzed in this study. The datasets generated during the current studyare available in the Cancer Genome Atlas (TCGA) public dataset (https://portal.gdc.cancer.gov/) and the Gene-Expression Omnibus (GEO) public dataset (https://www.ncbi.nlm.nih.gov/geo/).

## Author Contributions

JF and ZY performed all analysis as well as wrote the manuscript. XM and MY participated in data analysis. All authors participated in reviewing the manuscript and approved the final version.

## Conflict of Interest

The authors declare that the research was conducted in the absence of any commercial or financial relationships that could be construed as a potential conflict of interest.

## Publisher’s Note

All claims expressed in this article are solely those of the authors and do not necessarily represent those of their affiliated organizations, or those of the publisher, the editors and the reviewers. Any product that may be evaluated in this article, or claim that may be made by its manufacturer, is not guaranteed or endorsed by the publisher.

## Abbreviations

CNV, copy number variation; DC, dendritic cell; DEGs, differentially expressed genes; EMT, epithelial-mesenchymal transition; GEO, Gene-Expression Omnibus; GSVA, Gene set variation analysis; HR, hazard ratios; ICB, immunological checkpoint blockade; m^6^A, N6-methyladenosine; Pan-F-TBRS, Pan-fibroblast TGFb response signature; PCA, principal component analysis; ssGSEA, single-sample gene-set enrichment analysis; TCGA, The Cancer Genome Atlas; IPS, immunophenoscore; TIDE, tumor immune dysfunction and exclusion; TIME, tumor immune microenvironment.
