# Supplementary material for: Comprehensive Analysis of m6A Regulators Characterized by the Immune Cell Infiltration in Head and Neck Squamous Cell Carcinoma to Aid Immunotherapy and Chemotherapy
Source: Front Oncol. 2021 Nov 29;11:764798. doi: 10.3389/fonc.2021.764798 (PMC8670405; doi:10.3389/fonc.2021.764798)
Supplement: Supplementary file 1 [file DataSheet_1.docx]

**Supplementary Figures**


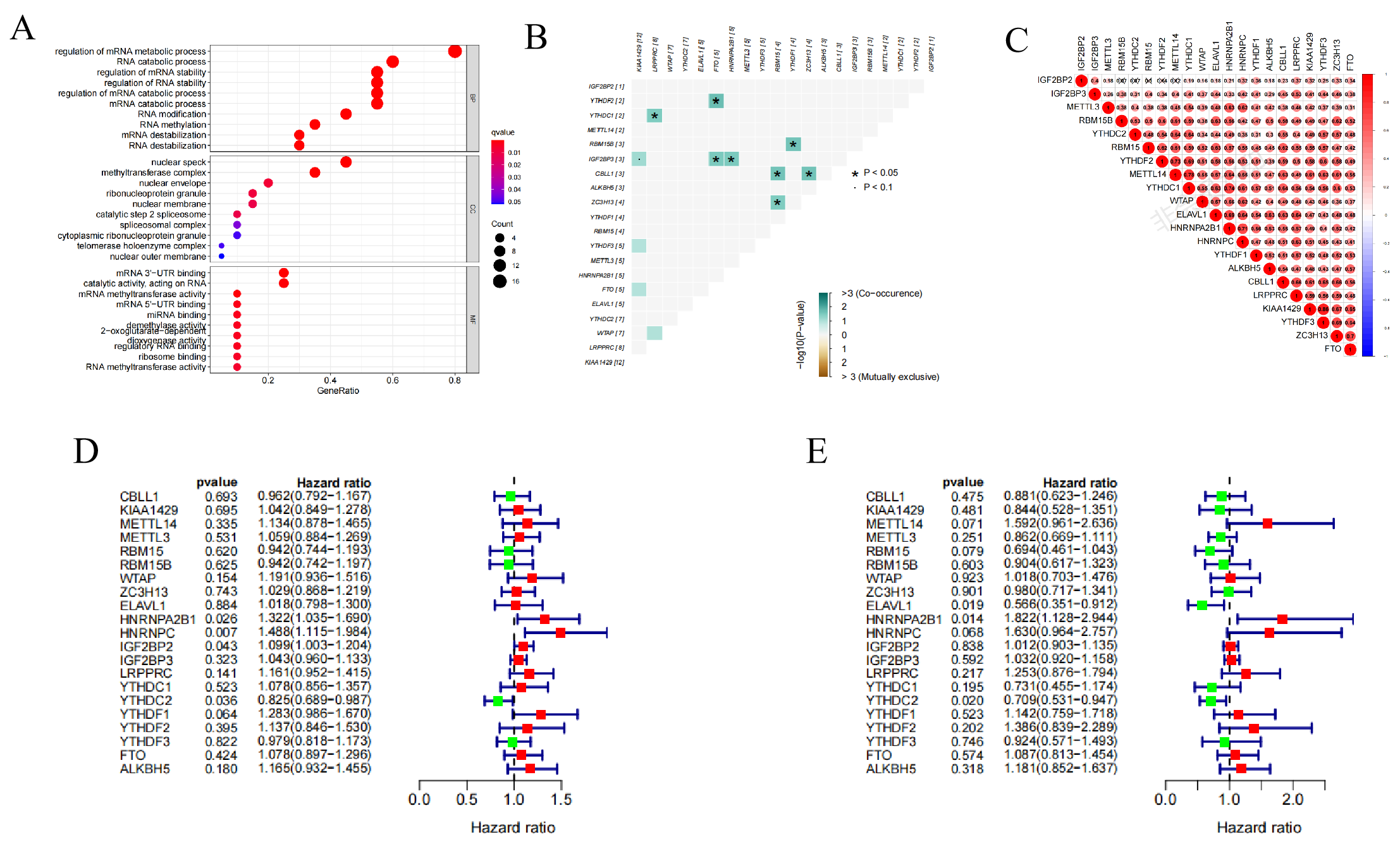


Figure S1. The correlation and prognostic results of the 21 m6A regulators.

(A) The GO enrichment of the 21 m6A regulators. (B) The mutation co-occurrence and exclusion analyses for 21 m6 A regulators. (C) Correlation between 21 m6 A regulators in HNSCC using the Spearman’s method. The subgroup analyses of 21 m6 A regulators in TCGA-HNSCC cohort by univariate Cox regression and multivariate Cox regression (E). Hazard ratio >1 represented risk factors and hazard ratio < 1 represented protective factors.


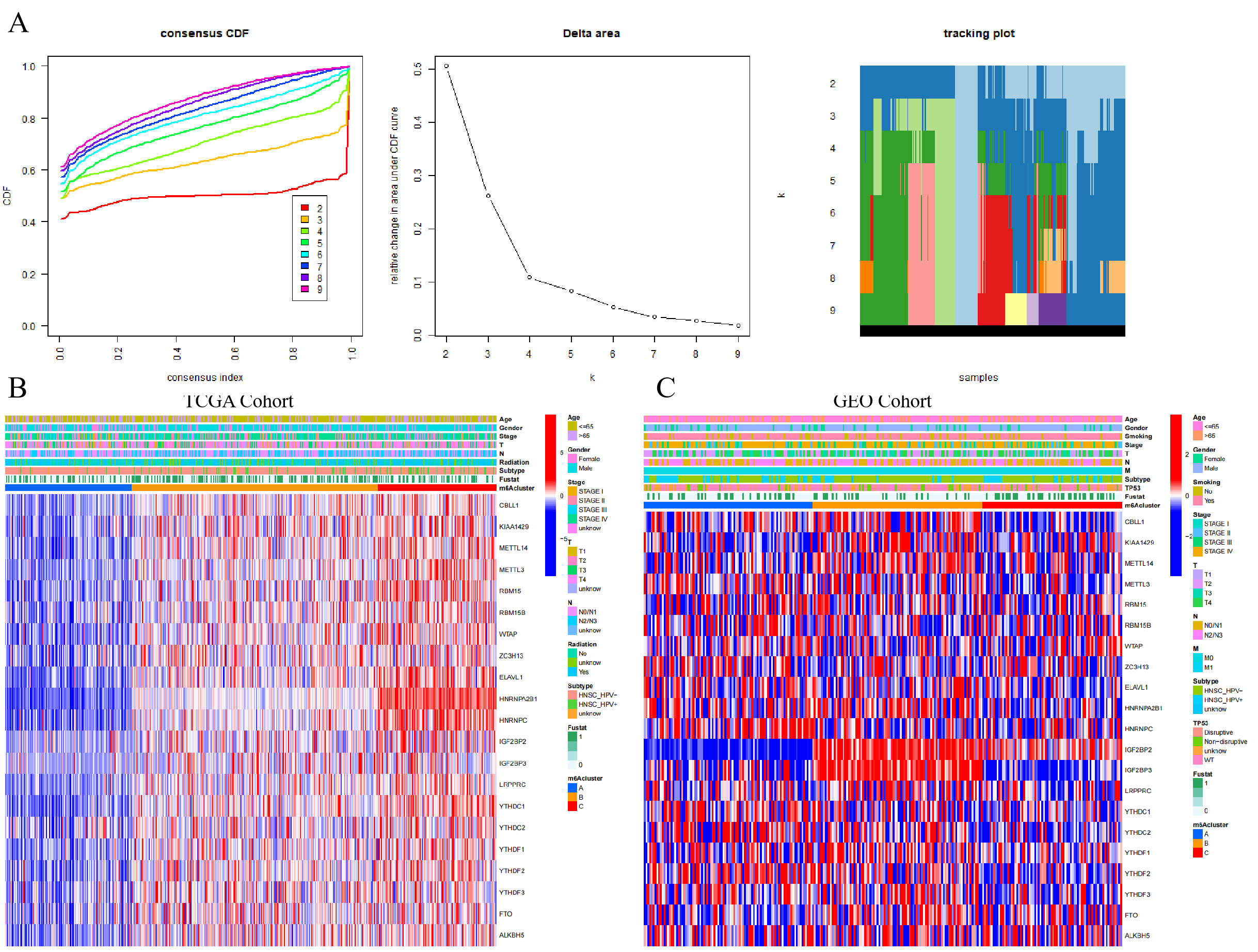


Figure S2. Consensus clustering of 21 m6 A regulators in the TCGA cohort.

(A) The cumulative distribution function (CDF) for k=2 to 9 in right panel; relative change in area under the CDF in middle panel; tracking plot in left panel. (B-C) The results of heatmap in the TCGA cohort (B) and GEO cohort (C).


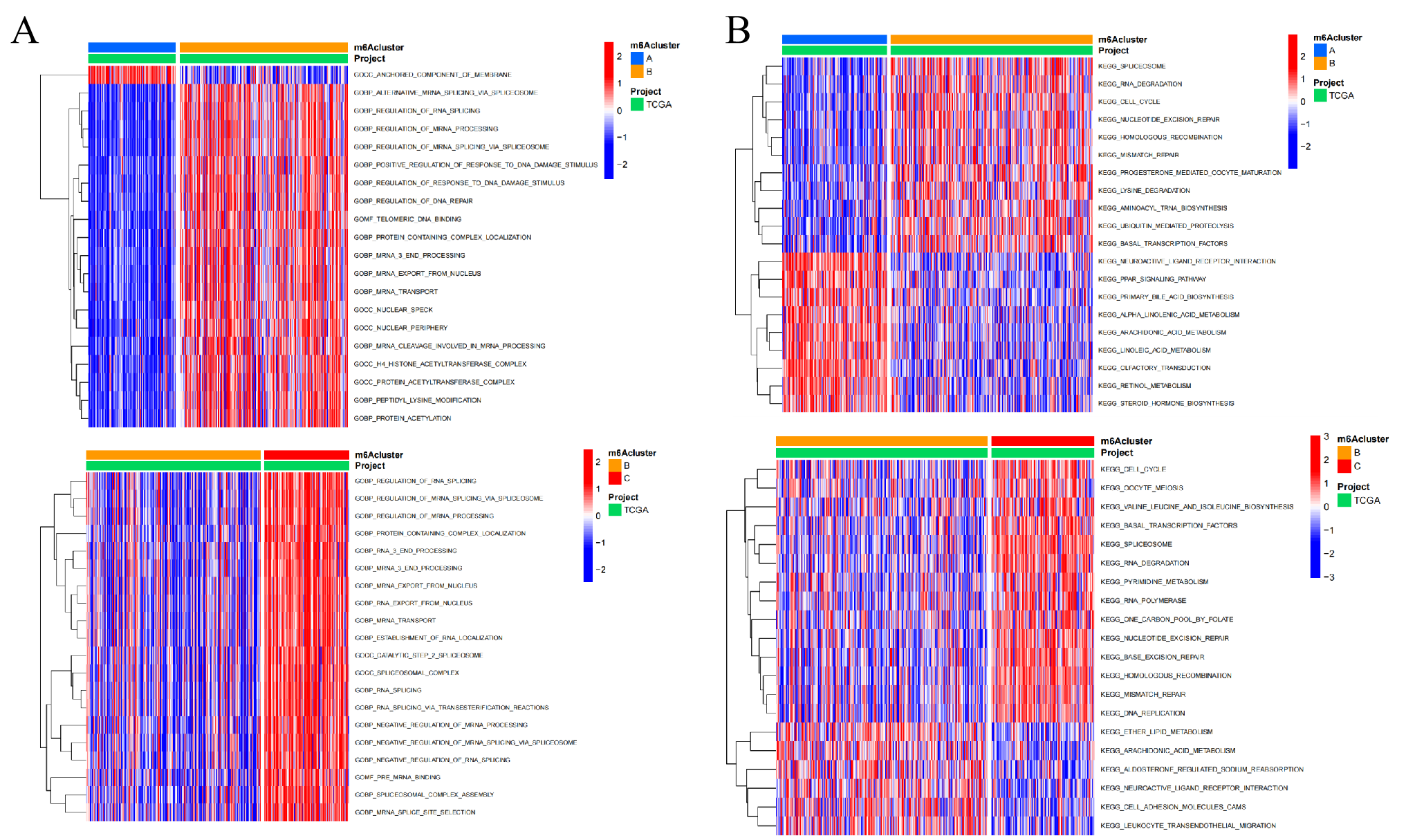


Figure S3. The biological functions and pathways of each m6A clusters.

(A-B) GSVA showed the biological pathways in each m6A clusters. The heatmap was used to visualize these biological functions and pathways. (A) GO enrichment analysis; (B) KEGG enrichment analysis.

##
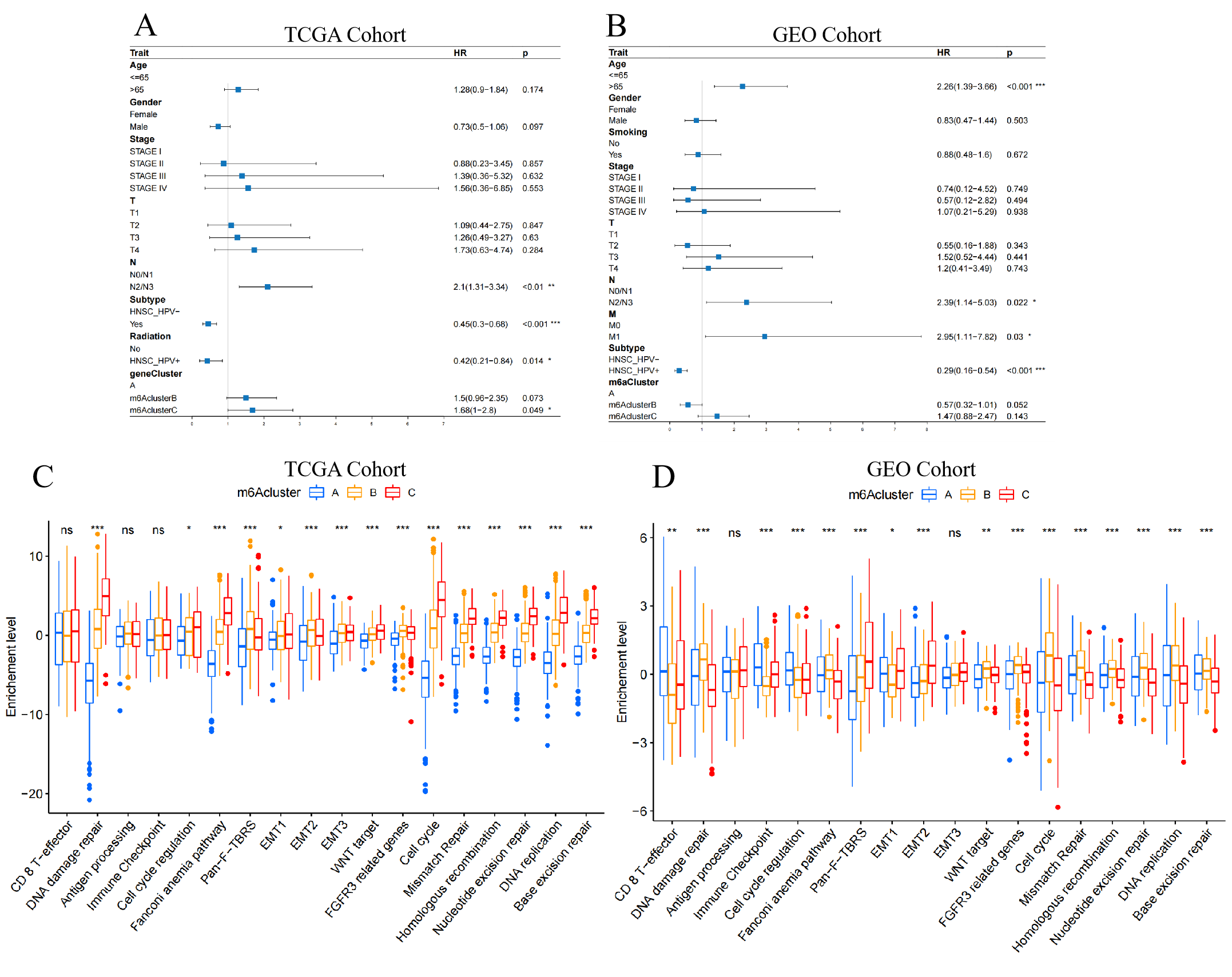


Figure S4. The prognostic value and enrichment of known signatures in three m6A clusters.

(A-B) The multivariate Cox regression estimated clinical prognostic value of m6A clusters in The TCGA cohort (A) and GEO cohort (B). The difference of known signatures including stromal-related signatures, tumor-related signatures and immune-related signatures among three m6A clusters in the TCGA (C) and GEO (D) cohort.


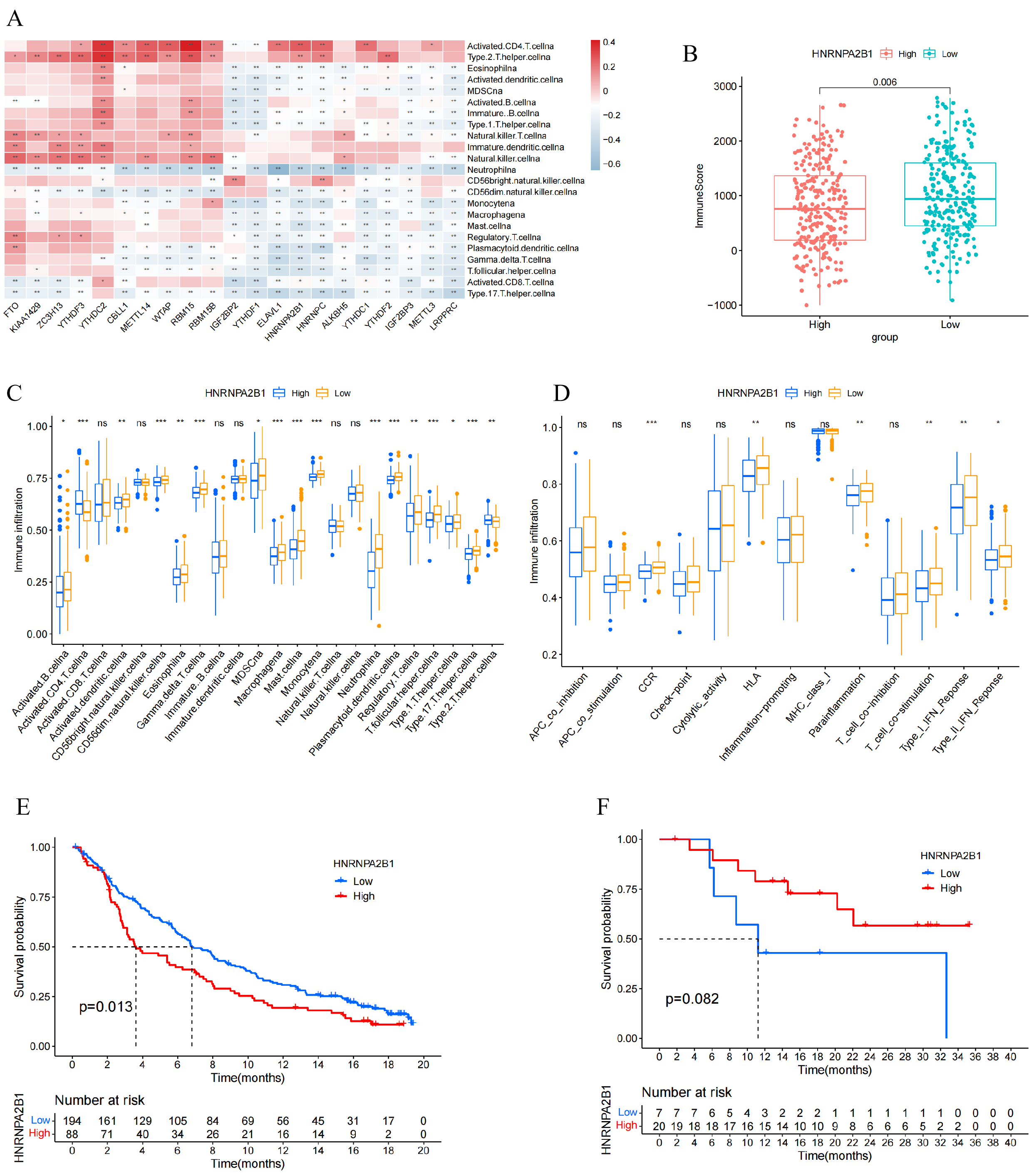


Figure S5. The correlation between infiltration immune cells and m6 A regulators and the roles of HNRNPA2B1 in HNSCC.

(A) The correlation between each immune cell type and each m6 A regulator using Spearman’s method. (B-D) The difference in the distribution of each immune cell (B) , ImmuneScore (C) and ImmuneFunctions (D) between HNRNPA2B1 high and low expression subgroups.(E-F) Kaplan-Meier survival curves presented patients with low and high expression of HNRNPA2B1 in the two anti-PD-L1 cohorts.

##
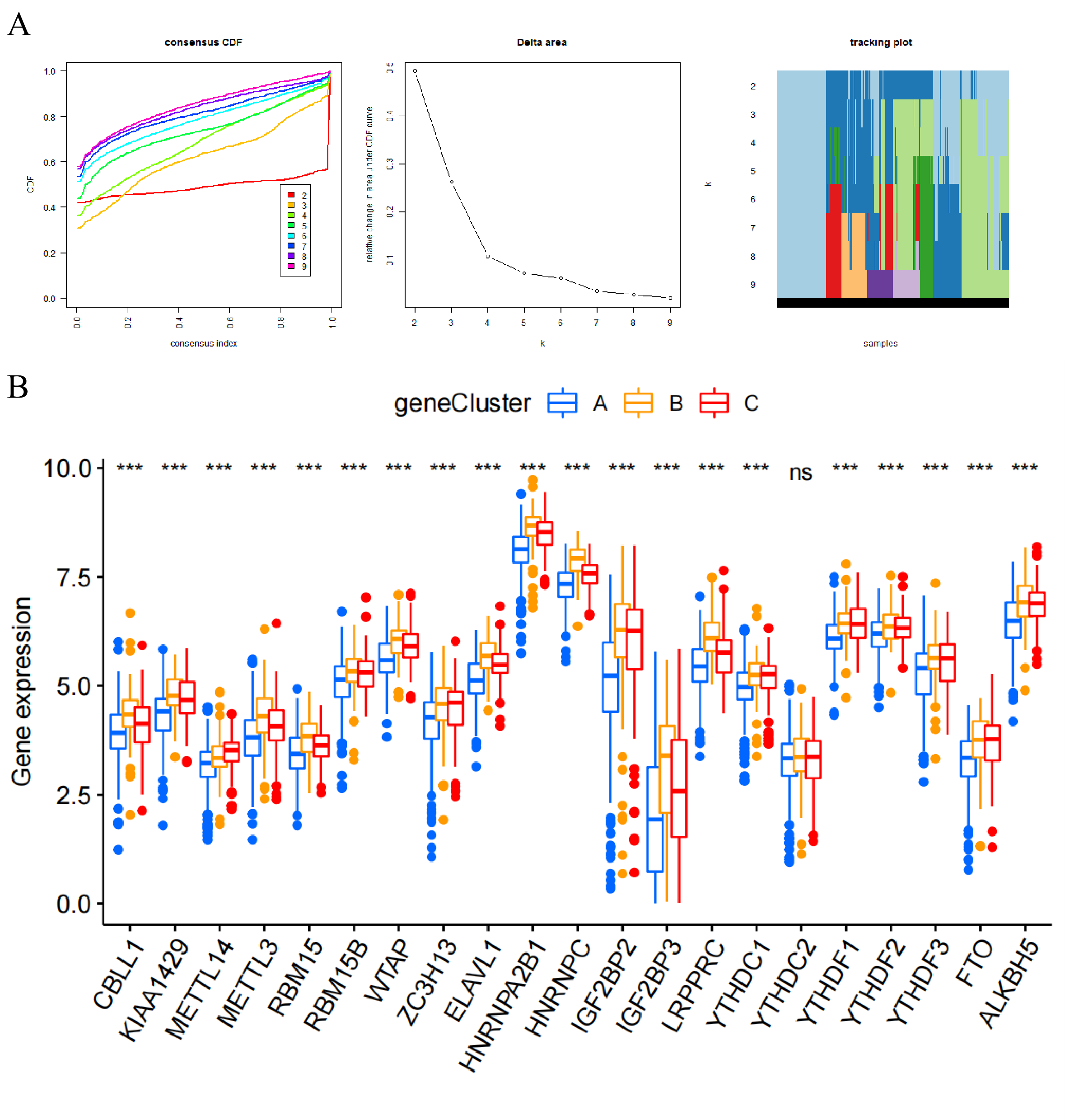


Figure S6. Consensus clustering of 311 m6Aclusters-related genes in the TCGA cohort.

(A) The cumulative distribution function (CDF) for k=2 to 9 in right panel; relative change in area under the CDF in middle panel; tracking plot in left panel. (B) The result of heatmap in the TCGA cohort.


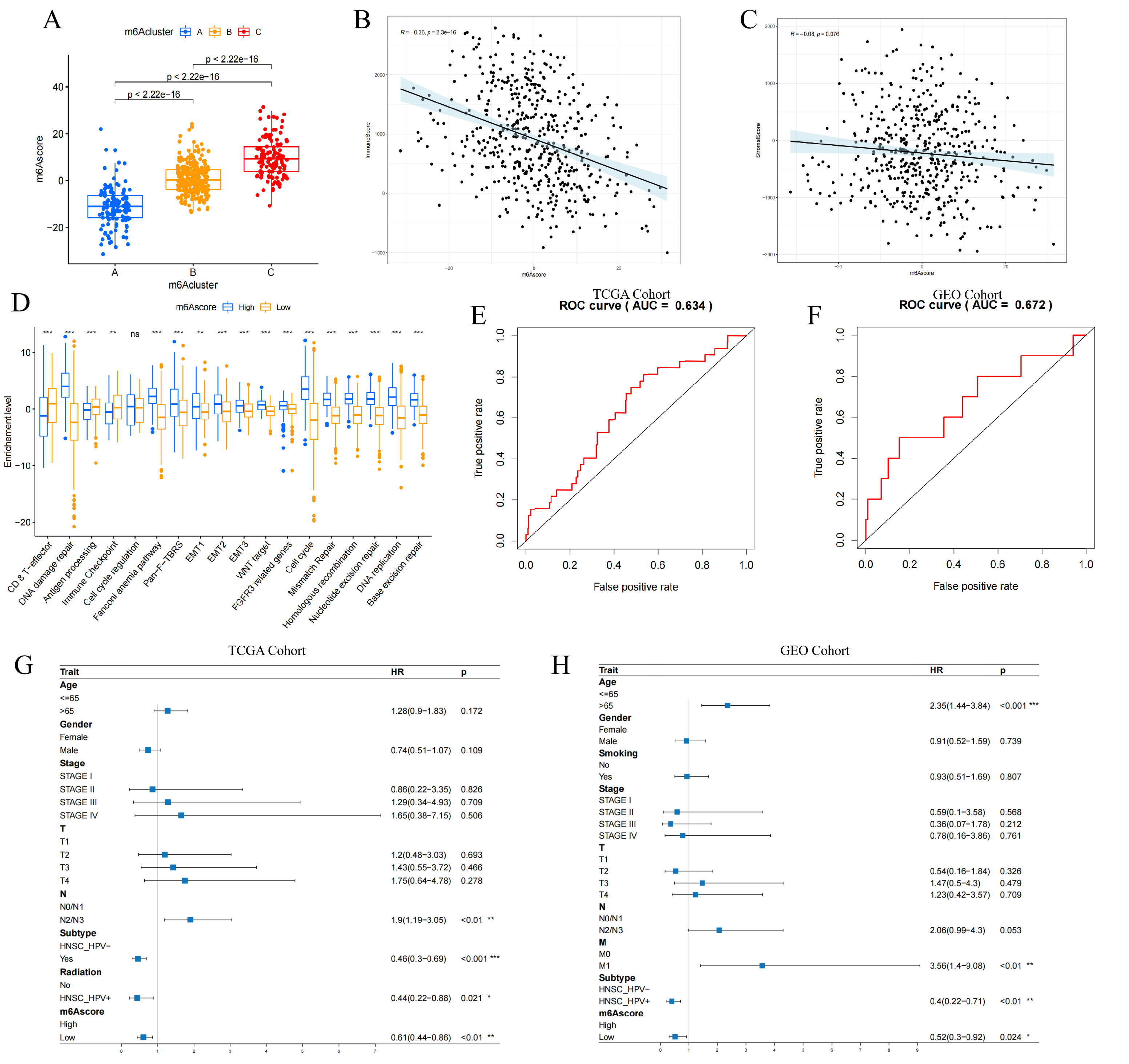


Figure S7. The m^6^A score associated with immune-related score and survival prognosis. (A) Differences in m^6^A score among three m6Aclusters. (B-C) Inverse correlation between the m^6^A score and the immuneScore, stromalScore. (D) Compared with the high m^6^A score subgroup, the low m^6^A score subgroup exhibited a higher proportion of suppressor cells and immune checkpoints. (E-F) The AUC of m^6^A score measured by ROC in TCGA cohort (E) and GEO cohort (F), AUC=Area under curve. (G-H) Multivariate Cox regression estimate the prognostic value of m^6^A score in TCGA cohort (G) and GEO cohort (H).


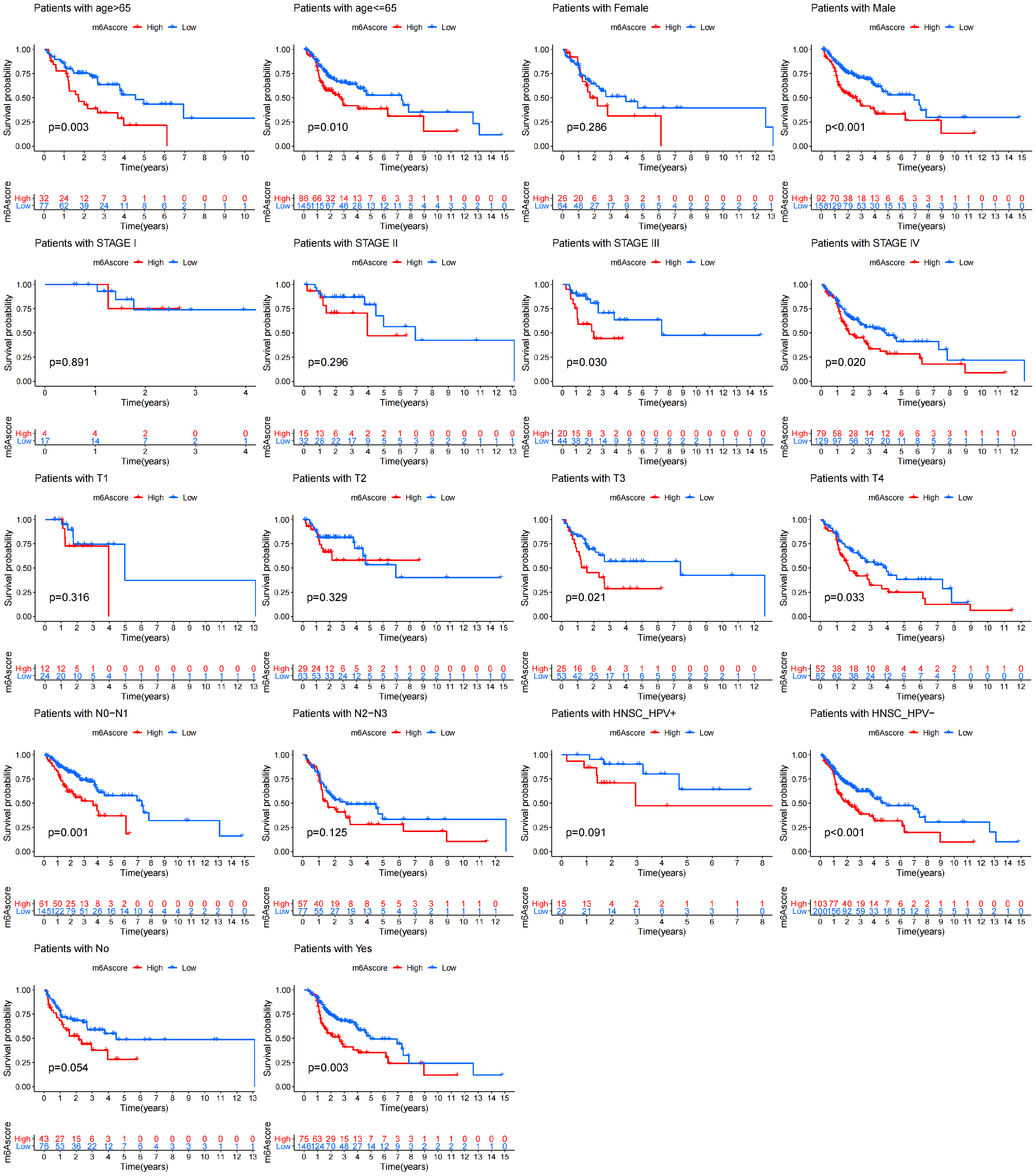


Figure S8. The Prognostic subgroup analysis of m^6^A score based on the TCGA cohort.


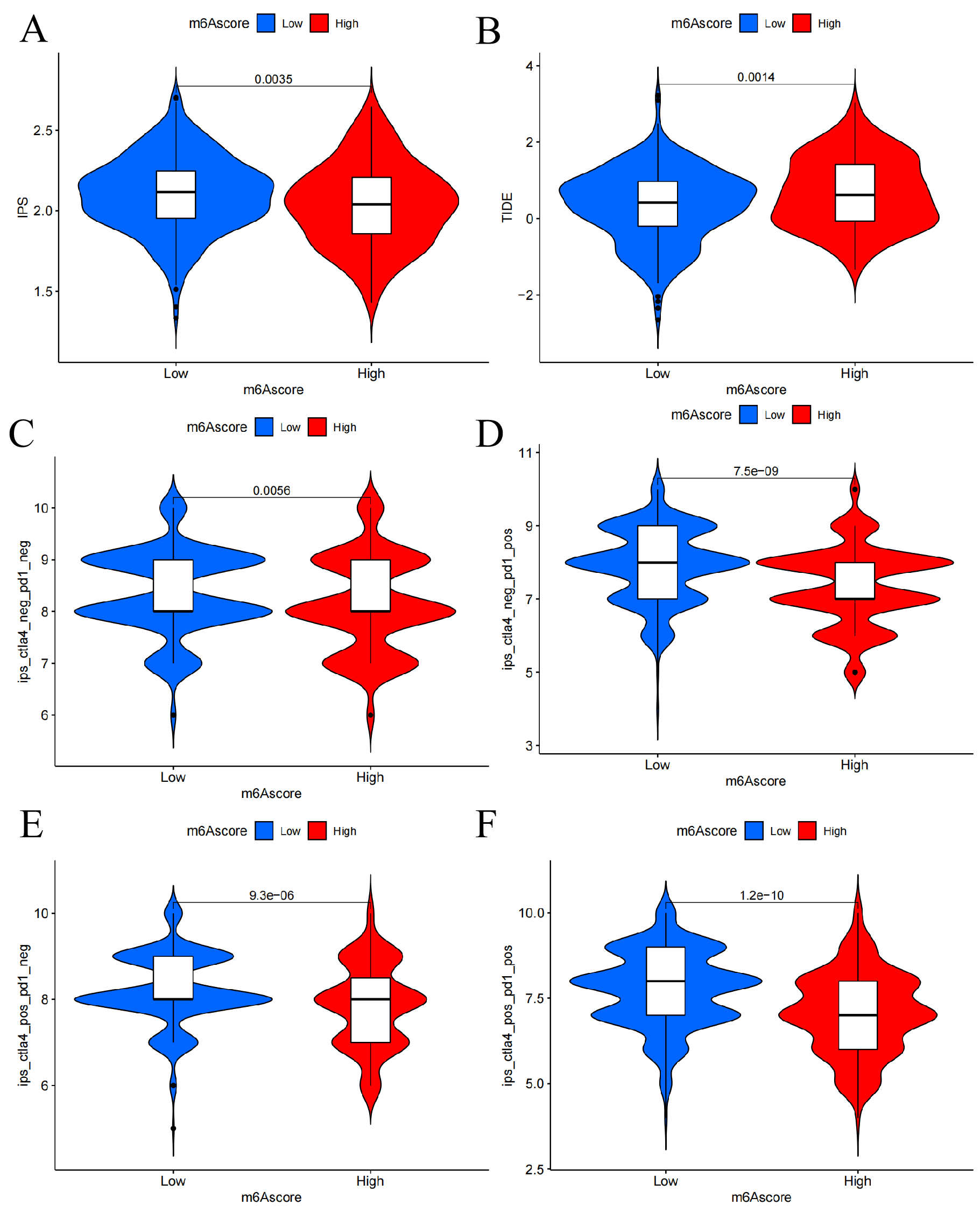


Figure S9. The m^6^A score predicts immunotherapeutic benefits. (A-B) The TIDE (A) and IPS (B) were compared between high and low m^6^A score subgroups in TCGA-HNSCC, respectively. (C-F) The difference between high and low m^6^A score subgroups stratified by CTLA-/PD1- (C), CTLA-/PD1+ (D) , CTLA+/PD1- (E) and CTLA+/PD1+ (F) in the TCGA cohort.


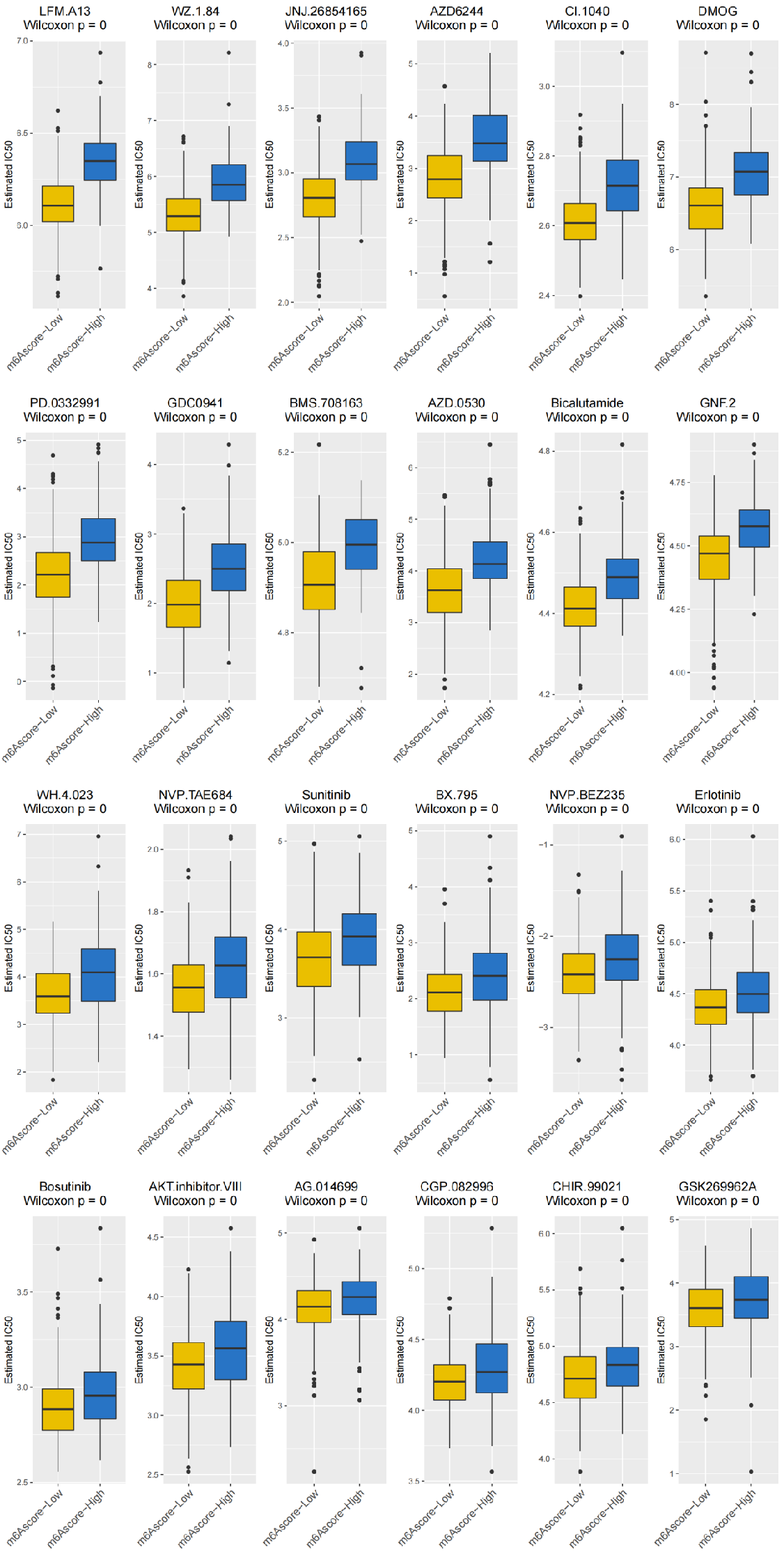


##
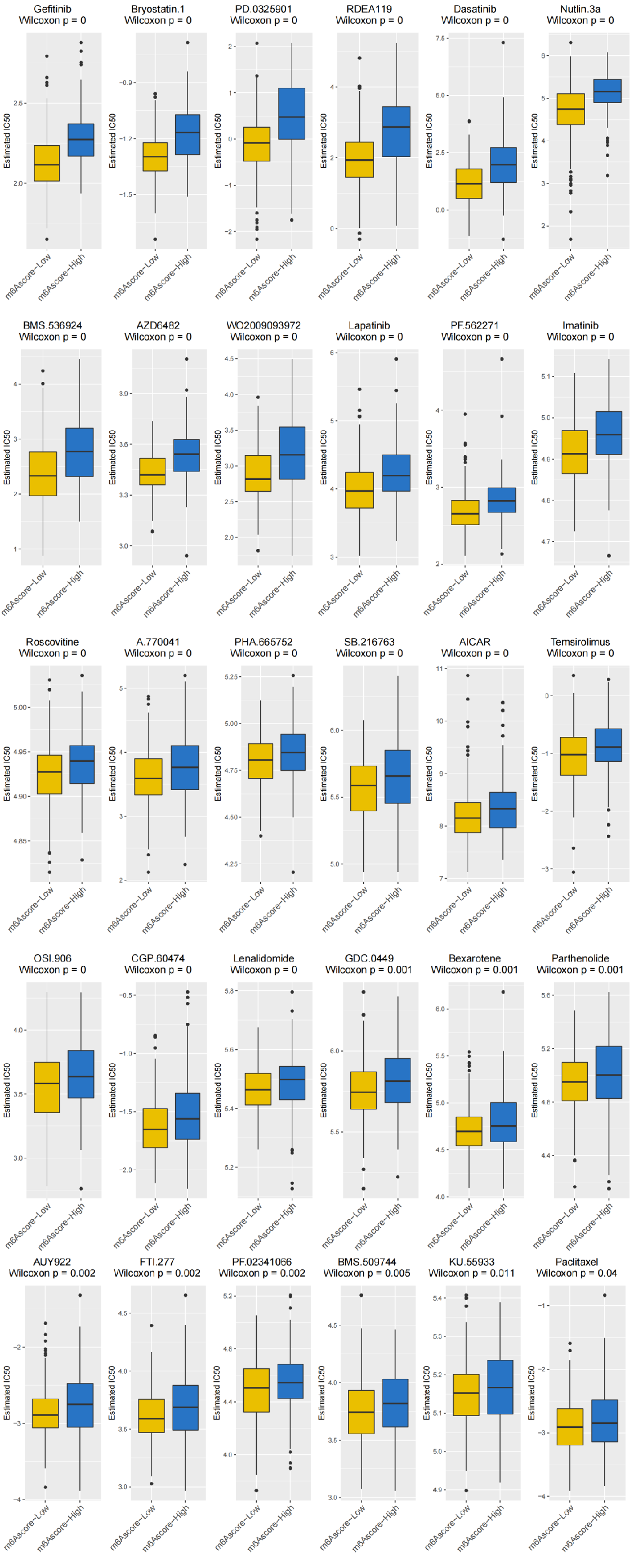


Figure S10. Box plots inferred IC50 of antitumor drugs between low and high m6Ascore.
